# Screen time and sleep among medical students in Germany

**DOI:** 10.1038/s41598-023-42039-8

**Published:** 2023-09-19

**Authors:** Lukas Liebig, Antje Bergmann, Karen Voigt, Erika Balogh, Béla Birkas, Nora Faubl, Theresa Kraft, Konrad Schöniger, Henna Riemenschneider

**Affiliations:** 1 Present Address: Department of General Practice, Medical Clinic 3, Faculty of Medicine Carl Gustav Carus, Technische Universität Dresden, Dresden, Germany; 2 Department of Public Health Medicine, University of Pécs Medical School, Pecs, Hungary; 3 Department of Behavioral Sciences, University of Pécs Medical School, Pecs, Hungary; 4 Institute for Medical Informatics and Biometry (IMB), Faculty of Medicine Carl Gustav Carus, Technische Universität Dresden, Dresden, Germany; 5 Department of Biological and Clinical Psychology, Institute of Psychology, Martin-Luther-Universität Halle-Wittenberg, Halle, Germany

**Keywords:** Medical research, Risk factors

## Abstract

Medical students are a vulnerable group for harmful health behaviours due to academic stress. Increased screen time is associated with adverse health behaviour, particularly delayed bedtime, shorter sleep duration and poorer sleep quality. This possible relationship has not yet been examined among medical students in Europe. Medical students at the Technical University of Dresden were invited to participate in an online questionnaire based cross-sectional study. To analyse correlations between screen time and sleep parameters, correlation coefficients, linear regression and mixed-model analysis were calculated. 415 students (average age 24 years, 70% female) were included in the analysis. The students reported an average of 7 h screen time per day and 7.25 h sleep duration per night. Approximately 23% (n = 97) reported sleeping less than 7 h per night and 25% (n = 105) reported fairly to very poor sleep quality. Students who reported more screen time for leisure went to bed significantly later (r = 0.213, p < 0.001). Students who spent more screen time for study/work tended to sleep shorter (r = − 0.108, p < 0.015). There was no significant association between screen time and sleep quality (p = 0.103). The results show a need for educational interventions to promote healthy sleep behaviour and to limit screen time.

## Introduction

Studying medicine is very challenging for students due to a high workload, time pressure, as well as a competitive environment. In particular, the amount of work, the difficulties in time management and maintaining a healthy lifestyle are a great burden for medical students^1–3^. The increased level of stress is associated with unhealthy behaviours in medical students. However, health-promoting behaviours are important both for one's own health and for performance during studies^4^. Various studies have already examined the health and risk behaviours of medical students^4–8^ but little research has been done on daily screen time and the associated changes in health behaviours among medical students in Europe. Studies indicate that increased screen time is associated with a variety of adverse physiological and psychological outcomes and is generally associated with poorer health behaviours^9^.

Screen time is defined as time spent on computers, tablets, smartphones, televisions or game consoles^10^. For young adults aged 18 and over, there is no guideline in Europe for a daily recommended duration of use. However, studies indicate that among adolescents, 4 to 5 h of daily screen time is associated with poor diet, obesity and physical inactivity^11–13^. A Canadian study found a significant association between screen time and the intensity of depressive symptoms and anxiety among adolescents^14^. In addition, a systematic review^15^ showed an adverse association between screen time and sleep parameters (specifically shorter sleep duration and delayed bedtime) in 90% of the studies. A negative association of sleep quality and the use of mobile devices has been showed in a meta-analysis^16^.

Sleep duration and sleep quality are related to cardiovascular^17^ and mental health^18^ but also to academic performance^19^ for which the time of sleep onset is also important^20^. Health and (academic) performance are important during the medical school and in the later career. There is evidence that the sleep quality of medical students is generally worse than that of students in other disciplines^21,22^. A meta-analysis published in 2020^22^ which evaluated 57 studies with 25.735 medical students worldwide, showed poor self-reported sleep quality in about 53% of the students (reference: Pittsburgh Sleep Quality Index (PSQI). Poor sleep quality was particularly common among medical students in Europe (prevalence: 65%). In addition, sleep duration among medical students worldwide (n = 4851) was reported to be 6.45 h per night on average^22^. In contrast, German medical students sleep significantly longer during the semester (7.80 h (± 0.95)^23^ or 8.22 h (± 0.14)^20^). These sleep times correspond to the American Academy of Sleep Medicine (AASM) guideline of regularly sleeping at least 7 h per night^24^. However, the interaction of screen time and sleep behaviour among medical students in Europe is largely unknown.

Therefore, the aim of the current study is to describe the daily screen time and sleep behaviour of medical students in Germany. In addition, it will be examined whether increased screen time is associated with a later bedtime, shorter sleep duration or poorer sleep quality.

## Methods

The multicentre cross sectional study “Medical Student Health Survey” was conducted in collaboration with the Department of General Practice at the Faculty of Medicine Carl Gustav Carus at the Technische Universität Dresden (TUD), the research group Applied medical psychology and medical sociology of the University Hospital Carl Gustav Carus at the TUD and the Departments of Public Health and Behavioural Sciences at the University of Pécs (UP), Hungary. The questionnaire used in the previous studies^5,25,26^ was developed further and adapted for the online LimeSurvey application (version 2.50+). The medical students at all study sites were invited to participate voluntarily and anonymously as part of the multicentre cross-sectional study. Informed consent was obtained from all students included in this study. Only data from TUD students in Germany were considered for this analysis. The invitation to participate in the online survey at the TUD was made through various channels such as e-mail invitations by the Student Council of Medicine and the Teaching Unit at the TUD, digital advertising in social media, but also by project staff in the context of lectures. The survey was conducted from January to July 2020. During that time, the students were affected by lockdowns and online classes due to the Covid-19 pandemic. In order to minimise potential influences of these prevention measures, which were considered temporary at the time, the recruitment was paused between March and May 2020.

The online questionnaire, which consisted of a variety of standardised and self-developed questions, was pre-tested for validity and reliability. The survey contained over 110 items and covered 10 topics. In addition to socio-demographic data, it primarily recorded the health status (general health, mental health, vaccination status, etc.) and health behaviours (sleep, diet, physical activity, media consumption, etc.) of medical students.

Screen time spent daily on the computer/tablet PC/mobile phone was assessed using self-generated items that captured time spent for both study/work and leisure (browsing/social media/games/videos etc.) (in hours). The specified screen time for study/work and leisure was added up to calculate a new variable: total screen time (TST). The questions on sleep behaviour were selected from the Pittsburgh Sleep Quality Index (PSQI). The present analysis focused on the following questions:

1. Bedtime: During the past month, what time have you usually gone to bed at night? Answer option: *Time*
2. Sleep duration: During the past month, how many hours of actual sleep did you get at night? (This may be different than the number of hours you spent in bed.) Answer option: *Time in hours*
3. Sleep quality: During the past month, how would you rate your sleep quality overall? Answer options: *Very good (0), Fairly good(1) , Fairly poor (2), Very poor (3)*
4. Time of getting up: During the past month, what time have you usually gotten up in the morning? Answer option: *Time.*

The standardisation of subjective sleep quality (Question 3.) via PSQI allows the calculation of a mean value and a comparison with other studies.

Since various determinants may be related to the daily-spent screen time and the sleep behaviour^27,28^ the following factors were taken into account: socio-demographic data (age, gender), the existence of a relationship or children, the living situation and data on physical activity and BMI. While the questions on the existence of a relationship or own children could be answered with yes or no, information on the living situation was dichotomised into living alone vs. not living alone. Being physically active (vs. inactive) was defined by physical activity 2 or more days a week to the point of sweating or being out of breath. In addition, BMI was calculated based on self-reported height and weight, and classification into underweight (BMI < 18.5), normal weight (BMI between 18.5 and 24.9), overweight (BMI between 25 and 29.9) and obese (BMI ≥ 30) was made based on the WHO guideline.

Statistical analyses were conducted using IBM SPSS 28.0. Group-specific differences in mean values of metric data were tested for significance using a t-test for independent samples. Group-specific differences in means of ordinal scaled data were tested for significance with a chi^2^-test. Group-specific differences in means between more than 3 groups were calculated with a one-factor analysis of variance (ANOVA).

Pearson's correlation coefficient, mixed model analysis, linear regression and partial correlation were calculated to determine the associations between screen time and bedtime, and between screen time and sleep duration. The test for a correlation between screen time spent and sleep quality was performed using Spearman's Rho (non-parametric test).

### Ethics approval

This study was performed in line with the principles of the Declaration of Helsinki. Approval was granted by the Ethics Committee of the Technische Universität Dresden (Date 16/12/2019; No. EK 15012014).

## Results

### Study population

With 476 completed questionnaires from 1418 enrolled medical students at the TUD, the response rate was approx. 34%. Of the 476 questionnaires only responses from students who reported screen time (for study/work and leisure) were included (n = 417). Implausible data on bedtime (n = 2, example: 11:30 a.m.) were transformed into plausible values (11:30 p.m.). In addition, data of students who reported a total screen time and sleep time of 24 h or more (n = 2) were excluded. The sample had an average ge of 24.4 years (SD = 3.9) and was approximately 70% (n = 292) female. Approximately 1/3 of the students (35%) were in pre-clinical semesters (1st–4th semester), whereas approximately 2/3 of the participating students were in clinical semesters (Table 1).

**Table 1:** Study population and screen time.

	Total n = 415	Screen time in h M (SD)		
		Study/work	Leisure	Total screen time (TST)
Age, M (SD)	24.4 (3.9)	4.7 (2.28)	2.24 (1.29)	6.94 (2.47)
Gender, n (%)				
Men	114 (27.5)	4.29 (2.32)	2.62 (1.64)	6.92 (2.63)
Women	292 (70.4)	4.86 (2.25)	2.08 (1.09)	6.95 (2.39)
Not reported, diverse	9 (2.2)			
t-test^a^		p = 0.024*	p < 0.001**	p = 0.916
Semester, n (%)				
Preclinical	142 (34.6)	4.89 (2.26)	2.13 (1.14)	7.02 (2.37)
Clinical	269 (65.4)	4.57 (2.29)	2.28 (1.33)	6.86 (2.47)
t-test		p = 0.172	p = 0.234	p = 0.511
Committed relationship, n (%)				
Yes	266 (64.7)	4.69 (2.13)	2.18 (1.29)	6.88 (2.39)
No	145 (35.3)	4.65 (2.54)	2.34 (1.29)	6.99 (2.60)
t-test		p = 0.859	p = 0.244	p = 0.656
Children, n (%)				
Yes	30 (7.3)	4.72 (2.77)	1.70 (1.35)	6.42 (3.03)
No	381 (92.7)	4.68 (2.25)	2.28 (1.28)	6.96 (2.41)
t-test		p = 0.925	p = 0.018*	p = 0.252
Housing situation, n (%)				
Alone	116 (28)	5.08 (2.51)	2.31 (1.21)	7.39 (2.53)
With others	297 (72)	4.53 (2.17)	2.21 (1.32)	6.74 (2.42)
t-test		p = 0.03*	p = 0.454	p = 0.016*
BMI, n (%)				
Underweight (< 18.5)	15 (3.7)	5.00 (2.68)	2.37 (1.08)	7.37 (2.74)
Normal (18.5–24.9)	330 (81.1)	4.60 (2.25)	2.21 (1.30)	6.81 (2.45)
Overweight (25.0–29.9)	48 (11.8)	5.25 (2.42)	2.36 (1.35)	7.60 (2.46)
Obese (≥ 30)	14 (3.4)	4.50 (2.02)	2.50 (1.09)	6.93 (2.46)
ANOVA		p = 0.291	p = 0.739	p = 0.185
Physical activity, n (%)				
Inactive	96 (23.1)	4.73 (2.47)	2.24 (1.30)	6.97 (2.59)
Active	319 (76.9)	4.69 (2.23)	2.23 (1.28)	6.93 (2.43)
t-test		p = 0.882	p = 0.490	p = 0.440

*M* mean, *SD* standard deviation, *h* hours.

*Significant p < 0.05.

**Significant p < 0.001.

^a^Difference between genders (men/women).

### Screen time

On average, students reported spending almost 7 h per day in front of a screen (TST = 6.94 h (SD = 2.3)). On average, 4.70 h (SD = 2.3) of screen time was spent for study/work and 2.24 h (SD = 1.3) for leisure. The shortest screen time (TST) was 1.50 h, the longest screen time (TST) was 15 h per day.

The t-test for independent samples showed significant gender differences for screen time spent during the day. Female students reported significantly more screen time for study/work than male (M = 4.86 vs. M = 4.29, p = 0.024) and male students reported significantly more screen time for leisure than female (M = 2.62 vs. M = 2.08, p < 0.001). The resulting total screen time, however, did not differ significantly between genders. Students with children reported significantly less screen time for leisure than students without children (M = 1.70 vs. M = 2.28, p = 0.018). Students who lived alone reported significantly more time in front of a screen for study/work (M = 5.08 vs. M = 4.53, p = 0.03) and had significantly higher total screen time than those not living alone (M = 7.39 vs. M = 6.74, p = 0.016). Depending on potential influencing factors, such as semester, relationship, BMI and physical activity, there were no significant group differences in daily screen time (Table 1).

### Bedtime

The reported bedtime of the medical students (n = 415) varied from 9:00 p.m. to 3:00 a.m. The mean bedtime was 11:11 pm (SD = 1:00). Male students reported going to bed 15 min later on average than female students (p = 0.025). Depending on socio-demographic data such as housing situation, children, relationship and potential influencing factors such as semester, BMI and physical activity, there were no significant group differences in the mean bedtime (Table 2). The cumulative bedtimes by gender are shown in Appendix 1.

**Table 2:** Bedtime.

		Bedtime M (SD)
Total (n = 415)		11:11 pm (1:00)
Gender, n (%)		
Men	114 (27.5)	11:22 pm (1:00)
Women	292 (70.4)	11:07 pm (1:00)
Not reported, diverse	9 (2.2)	
t-test^a^		p = 0.025*
Semester, n (%)		
Preclinical	142 (34.2)	11:15 pm (0:58)
Clinical	269 (64.8)	11:09 pm (1:02)
t-test		p = 0.440
Committed relationship, n (%)		
Yes	266 (64.1)	11.10 pm (0:59)
No	145 (34.9)	11.15 pm (1:03)
t-test		p = 0.365
Children, n (%)		
Yes	30 (7.2)	11.04 pm (1:06)
No	381 (91.8)	11.12 pm (1:00)
t-test		p = 0.466
Housing situation, n (%)		
Alone	116 (27.9)	11.16 pm (1:01)
With others	297 (71.6)	11.10 pm (1:00)
t-test		p = 0.375
BMI, n (%)		
Underweight (< 18.5)	15 (3.6)	11:40 pm (1:13)
Normal (18.5–24.9)	330 (79.5)	11:10 pm (1:00)
Overweight (25.0–29.9)	48 (11.6)	11:16 pm (0:59)
Obese (≥ 30)	14 (3.4)	11:25 pm (1:11)
ANOVA		p = 0.215
Physical activity, n (%)		
Inactive	96 (23.1)	11:14 pm (1:01)
Active	319 (76.9)	11:10 pm (1:00)
t-test		p = 0.571

*M* mean, *SD* standard deviation.

*Significant p < 0.05.

**Significant p < 0.001.

^a^Difference between genders (men/women).

### Sleep duration

The medical students (n = 410) reported sleeping an average of 7.25 h (SD = 1.00) per night, with sleep times ranging from 4 to 10 h. Approximately 23% (n = 97) of the students reported sleeping less than 7 h per night. Students with children (n = 29) reported sleeping on average one hour less per night than students without children (M = 6.38 (SD = 0.9) vs. M = 7.32 (SD = 0.9), p < 0.001). Sleep duration did not differ significantly depending on gender, semester, relationship, children, housing situation, BMI and physical activity. (Table 3).

**Table 3:** Sleep duration.

		Sleep duration in h M (SD)
Total (n = 410)		
Gender, n (%)		
Men	113 (27.6)	7.16 (1.04)
Women	288 (70.2)	7.27 (1.00)
Not reported, diverse	9 (2.2)	
t-test^a^		p = 0.329
Semester, n (%)		
Preclinical	141 (34.0)	7.16 (1.02)
Clinical	265 (63.9)	7.29 (1.00)
t-test		p = 0.197
Committed relationship, n (%)		
Yes	261 (63.7)	7.30 (1.05)
No	145 (35.4)	7.17 (0.93)
t-test		p = 0.251
Children, n (%)		
Yes	29 (7.1)	6.38 (0.94)
No	377 (92.0)	7.32 (0.98)
t-test		p < 0.001**
Housing situation, n (%)		
Alone	114 (27.5)	7.19 (1.05)
With others	294 (71.7)	7.27 (0.99)
t-test		p = 0.458
BMI, n (%)		
Underweight (< 18.5)	15 (3.7)	7.20 (0.88)
Normal (18.5–24.9)	327 (79.8)	7.28 (0.95)
Overweight (25.0–29.9)	46 (11.2)	7.04 (1.27)
Obese (≥ 30)	14 (3.4)	6.96 (1.31)
ANOVA		p = 0.337
Physical activity, n (%)		
Inactive	95 (23.2)	7.20 (1.18)
Active	315 (76.8)	7.26 (0.95)
t-test		p = 0.585

*M* mean, *SD* standard deviation.

*Significant p < 0.05.

**Significant p < 0.001.

^a^Difference between genders (men/women).

### Sleep quality

Approximately 75% of the medical students (n = 309) reported having slept "fairly good" or "very good" in the last month. While 23% of medical students reported "fairly poor" sleep quality, about 2% reported "very poor" sleep quality (see Appendix 2). Even though all students with "very poor" sleep quality (n = 10) were female, no significant gender differences could be found with regard to sleep quality (p = 0.255). The average subjective sleep quality was 1.11 (SD = 0.69) (0 = very good to 3 = very poor).

There were significant differences between pre-clinical semesters and clinical semesters (p = 0.02) as well as between physically active vs. inactive medical students (p = 0.033) regarding sleep quality (Table 4). Whereas 33% of students in preclinical semesters reported very poor or fairly poor sleep quality (M = 1.21, SD = 0.68), only 22% in clinical semesters did so (M = 1.06, SD = 0.70). Very poor or fairly poor sleep quality affected 33% of physically inactive students (M = 1.24, SD = 0.77) compared to 24% of physically active students (M = 1.07, SD = 0.67).

**Table 4:** Sleep quality.

		Sleep quality M (SD)
Total (n = 414)		
Gender, n (%)		
Men	114 (27.5)	1.06 (0.64)
Women	291 (70.3)	1.14 (0.72)
Not reported, diverse	9 (2.2)	
t-test^a^		p = 0.303
Semester, n (%)		
Preclinical	142 (34.3)	1.21 (0.68)
Clinical	268 (63.73)	1.06 (0.70)
t-test		p = 0.040*
Committed relationship, n (%)		
Yes	265 (64.0)	1.11 (0.69)
No	145 (35.0)	1.12 (0.70)
t-test		p = 0.955
Children, n (%)		
Yes	30 (7.2)	1.33
No	380 (91.8)	1.10
t-test		p = 0.073
Housing situation, n (%)		
Alone	116 (27.5)	1.18 (0.69)
With others	296 (71.7)	1.09 (0.69)
t-test		p = 0.220
BMI, n (%)		
Underweight (< 18.5)	15 (3.6)	1.07 (0.59)
Normal (18.5–24.9)	329 (79.5)	1.10 (0.69)
Overweight (25.0–29.9)	48 (11.6)	1.10 (0.66)
Obese (≥ 30)	14 (3.4)	1.57 (0.85)
ANOVA		p = 0.095
Physical activity, n (%)		
Inactive	96 (23.2)	1.24 (0.77)
Active	318 (76.8)	1.07 (0.67)
t-test		p = 0.038*
Fulfilment AASM guideline		
Yes	313 (75.6)	0.97 (0.62)
No	97 (23.4)	1.56 (0.72)
t-test		p < 0.001**

*M* mean, *SD* standard deviation.

*Significant p < 0.05.

**Significant p < 0.001.

^a^Difference between genders (men/women).

Students who did not meet the AASM guideline of recommended sleep duration (n = 97) had lower subjective sleep quality (M = 1.56, SD = 0.72) than students who met the AASM guideline (n = 313, M = 0.97, SD = 0.62, p < 0.001).

### Association between screen time and bedtime

**Figure 1:**
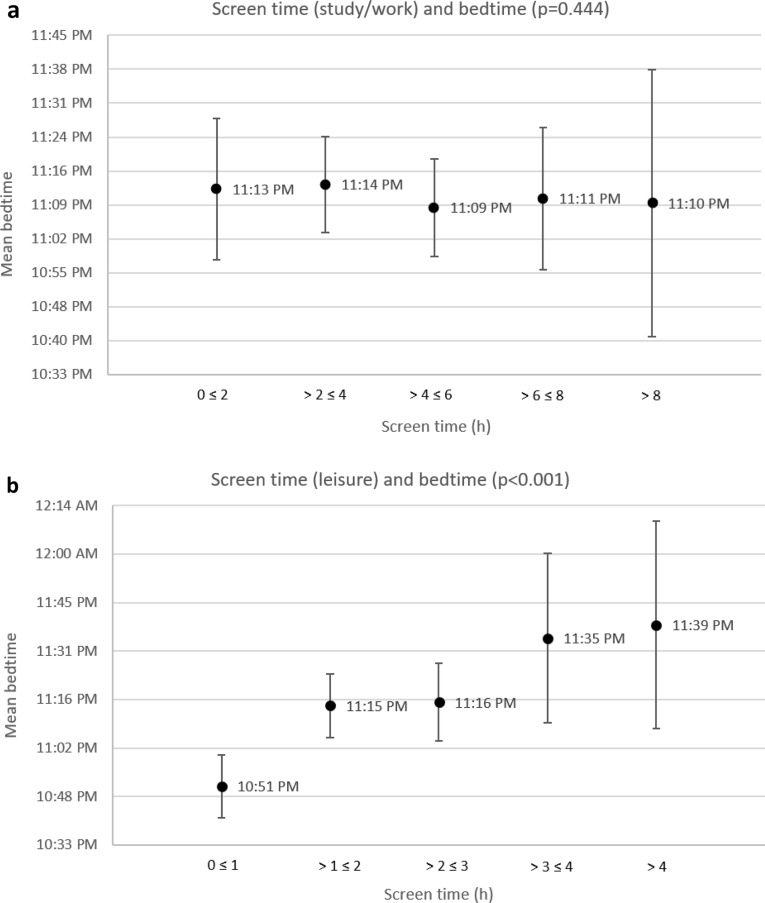
(**a**) Average bedtime according to the amount of screen time (study/work). (**b**) Average bedtime according to the amount of screen time (leisure). The error bars indicate 95% confidence intervals.

More screen time spent during the day was significantly associated with later bedtime among medical students. Most of this effect can be attributed to screen time spent for leisure (p < 0.001), as screen time spent studying/working was not significantly associated with bedtime (p = 0.444) (Table 5). This relationship is visualised in Fig. 1, which shows the average bedtime according to the amount of screen time. If the screen time (study/work and leisure) and the various influencing variables (gender, semester, relationship, children, living situation, BMI, physical activity) are considered in a mixed-model analysis, only the screen time spent for leisure is significantly (p < 0.01) associated with bedtime. A linear regression model showed that the students of the present cohort went to bed on average 10 min later for every hour they spent screen time for leisure (p < 0.001).

**Table 5:** Correlation coefficients between screen time and sleep parameters.

	Bedtime	Sleep duration	Sleep quality	Get-up time
Total screen time (TST)	0.105 p = 0.016*	− 0.122 p = 0.007*	0.065 p = 0.103	0.033 p = 0.253
Screen time study/work	− 0.007 p = 0.444	− 0.108 p = 0.015*	0.027 p = 0.291	− 0.111 p = 0.012*
Screen time leisure	0.213 p < 0.001**	− 0.042 p = 0.199	0.027 p = 0.292	0.260 p < 0.001**

*Significant p < 0.05, **significant p < 0.001.

### Association between screen time and sleep duration

**Figure 2:**
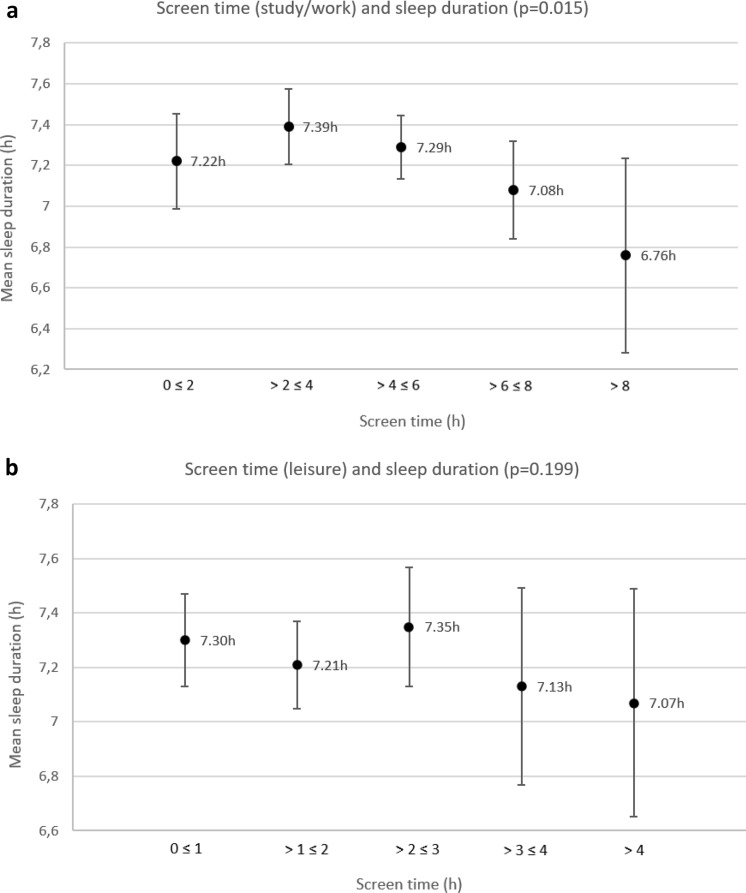
(**a**) Average sleep duration according to the amount of screen time (study/work). (**b**) Average sleep duration according to the amount of screen time (leisure). The error bars indicate 95% confidence intervals.

Total screen time spent during the day was significantly associated with sleep duration (r(408) = − 0.122, p = 0.007): The increased total screen time was associated with decreased sleep duration among medical students. Here, screen time spent for leisure was not significantly associated with sleep duration (p = 0.199), whereas screen time spent for study/work was significantly associated with sleep duration (p = 0.015) (Table 5). This relationship is visualised in Fig. 2, which shows the average sleep duration according to the amount of screen time. Based on the mixed-model analysis of screen time (study/work and leisure) and the various influencing variables (gender, semester, relationship, children, living situation, BMI, physical activity), there was a significant association of screen time spent on study/work (p = 0.038) and the existence of own children (p < 0.01) and sleep duration. However, a subsequent partial correlation showed that the correlation (effect size, p-value) of screen time (work/study) and sleep duration is independent of whether students have their own children.

### Association between screen time and sleep quality

Among the study sample, no correlation between screen time and sleep quality was found. There was no association with sleep quality either for total screen time (p = 0.103), for screen time spent for leisure (p = 0.292) or for screen time spent for study/work (p = 0.291) (Table 5).

### Association between screen time and get-up time

**Figure 3:**
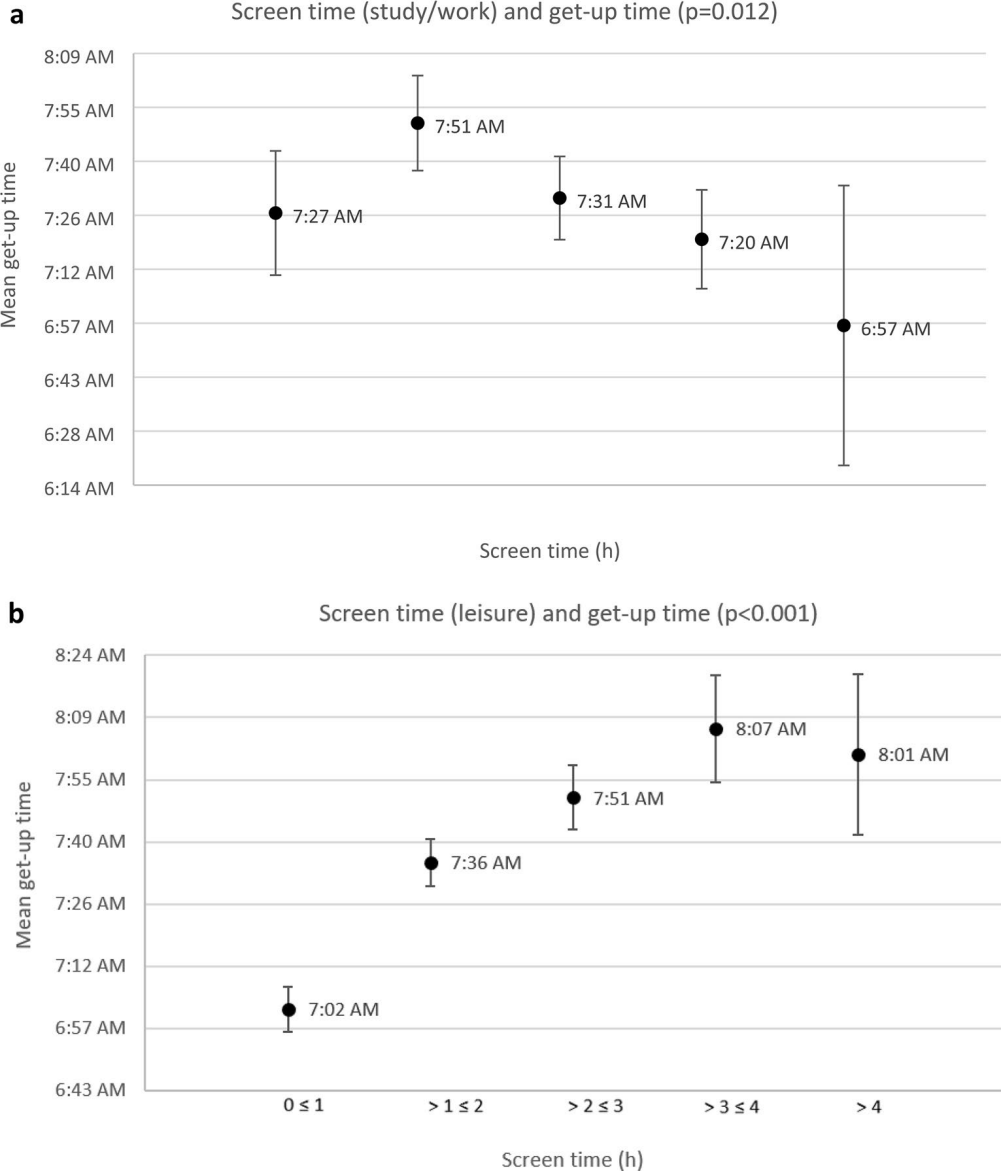
(**a**) Average get-up time according to the amount of screen time (study/work). (**b**) Average get-up time according to the amount of screen time (leisure). The error bars indicate 95% confidence intervals.

In the context of the present study, a significant association between screen time and get-up time was found. While TST was not associated with the get-up time (p = 0.253), the screen time spent for study/work was significantly negatively associated (p = 0.012), and the screen time spent for leisure was significantly positively associated (p < 0.001), with the time getting up (Table 5). This association is visualised in Fig. 3, which shows the average get-up time according to the amount of screen time.

## Discussion

### Screen time

The present study was able to provide an insight into the screen use and sleeping behaviour of medical students in Germany. On average, students spent about 7h per day in front of a screen, with male students spending more time in front of a screen for leisure and female students spending more time in front of a screen for study/work. Students with children spent significantly less screen time for leisure. Students living alone reported more screen time for study/work. Due to methodological differences, such as survey period (especially due to the Covid-19 pandemic), screen time by device and screen time by purpose of usage, a comparison of total screen time between studies with medical students is difficult. Compared with a study from India^29^, medical students at TUD reported a higher total screen time (5.13 h vs. 6.94 h per day). In comparison with Norwegian students of various disciplines (n = 48,184), medical students at the TUD spent about the same amount of time in front of a screen per day (on average 7–8 h vs. 6.94 h per day)^27^.

Nevertheless, making conclusions regarding screen time is difficult since there is no guideline for safe daily screen use for the age of the present cohort (young adults, ≥ 18 years). In Germany, the Federal Centre for Health Education (Bundeszentrale für gesundheitliche Aufklärung) recommends a maximum media use time of 45 to 60 min per day for children aged 6 to 10 years^30^. There is no guideline for the age group 10 years and older. However, there is an Australian guideline for screen time for 5–17 year olds, which recommends a daily usage time of < 2 h per day (excluding screen time for schoolwork)^31^. Nevertheless, the increased screen time due to home office and home schooling during the Covid-19 pandemic implies a need for new guidelines on screen time and associated health outcomes^32^.

### Sleep parameters

The medical students of the present study slept approximately 7.25 h per night. Approximately one in four medical students did not meet the AASM guideline of at least 7 h of sleep per night. In particular, students with children slept significantly less and 65% of this group were not able to meet the AASM guideline. The sleep duration of the present cohort is somewhat lower than the previously reported sleep duration (reference: PSQI) of medical students in Germany of 7.80 h^23^ and 8.22 h^20^. However, they exceed the international average sleep duration of medical students (n = 4851) reported in the meta-analysis by^22^ of 6.45 h of sleep per night. Nevertheless, it is unclear what explains the difference in sleep duration among medical students in different countries. In addition, the sleep duration reported in studies can also be influenced by various methodologically relevant factors such as variances in self-reported sleep parameters^33^, the season (winter vs. summer)^34,35^ and the section of the semester (before vs. after the exam period)^23^ and thus may help explain the small differences.

Approximately one in four students reported fairly poor or very poor sleep quality whereas physically active students (vs. inactive students) and students in clinical semesters (vs. pre-clinical semesters) as well as students who met the AASM guideline reported having better sleep quality. The average subjective sleep quality via PSQI was 1.11 (SD = 0.69). In a meta-analysis of Rao et al.^22^, including 41 studies with 16,748 medical students, the average score of subjective sleep quality was 1.22 (95% CI 1.04–1.41). This indicates that medical students in Germany have a slightly better subjective sleep quality than medical students in average. However, the data from Rao et al.^22^ also indicates that the subjective sleep quality is rated better than the sleep quality calculated by the PSQI total score. This speaks for a need for education on the topic of sleep quality, performance and health in medical studies.

### Correlation between screen time and sleep parameters

Our study showed that screen time is associated with the sleep behaviour of medical students. While the hypotheses that increased screen time is associated with a later bedtime as well as shorter sleep duration could be confirmed, no association between screen time and sleep quality was found. The associations between screen time and sleep parameters varied with the purpose of the screen use: while increased screen time for leisure was associated with a later bedtime (p < 0.001), screen time spent for study/work was associated with a shorter sleep duration (p = 0.015).

This could indicate that screen time spent for leisure tends to take place in the second half of the day (since the compulsory parts of study are primarily in the first half of the day) and thus increased screen time in the second half of the day chronologically shifts bedtime. At the same time, increased screen time for study/work does not lead to a later bedtime but to a shorter sleep duration. The analysis conducted on screen time and get-up time revealed that students with increased screen time for study and work tend to get up earlier (p = 0.012) and students who spend more screen time for leisure tend to sleep longer (p < 0.001). Both a later bedtime and a shorter sleep duration are associated with various negative outcomes. Genzel et al.^20^ showed in a cohort of German medical students, that students whose mean bedtime was earlier achieved significantly better grades than students who had a later sleep–wake rhythm. A later sleep–wake rhythm, also known as a late chronotype, can be caused by various factors such as genetics or lifestyle^36^. On the other hand, a late chronotype is associated with an increased risk for affective disorders^37,38^, anxiety disorders or substance abuse^38^. In addition, various diseases such as high blood pressure and type 2 diabetes^39^ as well as poorer nutrition^40^ are associated with a late chronotype. Another factor, which is often associated with a later bedtime, is poorer sleep quality^41,42^.

Shorter sleep duration is also associated with poorer academic performance^43,44^, poorer self-reported health^45,46^ and cardiovascular health^46^. Nevertheless, it must be emphasised that the correlation between sleep duration and screen time for study/work demonstrated in the present study is rather low, whereas, for example, the negative correlation of parenthood and sleep duration (p < 0.001) was stronger.

Even though the mixed-model analysis did not show an independent effect of gender on sleep parameters, it could be shown that male students spend significantly more screen time for leisure and female students spend significantly more screen time for study/work. In order to raise awareness for the association of screen time and sleep behaviour, acknowledging the gender differences among medical students is important.

No association between screen time and sleep quality was found in the present study. This is consistent with some previous studies^29,47^, contrasted to other studies that confirmed the association^16,28,33,48–50^. The contradictory results of the studies are possibly due to the timing^16,51^ and the type of screen (smartphone vs. TV)^52^. While screen use earlier in the day has no effect on sleep quality, screen use in the evening and night time hours is associated with poorer sleep quality^16,51^. These factors were not considered in the present study.

The correlations between screen time spent for leisure and later bedtime, and between screen time spent studying/working and shorter sleep duration, as shown in this study, highlight a need for awareness-raising on the topic of screen use and sleep behaviour. A later bedtime and shorter sleep duration are associated with various negative health consequences and poorer academic performance. The WHO also recommends in its guideline "Physical Activity and Sedentary Behaviour"^53^ that sedentary activities, such as TV watching or computer use, should be minimised, as they are associated with a variety of negative health outcomes. Furthermore, the WHO recommends replacing these sedentary activities with moderate to vigorous physical activity to reduce the negative consequences of increased sitting time. In the context of the present results, this recommendation argues for a reduction of screen time spent for leisure among medical students. Furthermore, since physically active students in the present cohort had a significantly better sleep quality than physically inactive students, a replacement by physical activity would be additionally beneficial.

### Limitations

The present study has some limitations. Due to its cross-sectional design, no statements on causalities can be made. Accordingly, a later bedtime could be due to a longer screen time—however, a genetically determined, later chronotype could also contribute to higher screen use in the evening. The available data are self-report data and thus susceptible to subjective bias. Previous studies indicate a discrepancy between objective and subjective data for both sleep^33^ as well as for screen^15^ behaviour. Furthermore, no calculation of the PSQI total score was possible since not all items of the instrument were included in the survey instrument. This makes comparability with other studies difficult and subjective bias more likely, especially with regard to sleep quality. The present data were collected during the first half of 2020. At that time, a temporary lockdown and further restrictive measures forced medical students in Germany to complete more study tasks from home. Studies indicate that students spent more screen time under these conditions than at other times^28,54^. However, at the time of the study, there were only few, temporary restrictions in Saxony, whereas restrictive measures from the second half of 2020 to the end of 2021 were more restrictive and had a higher impact on people’s behaviour.

## Conclusion and outlook

Many medical students, especially students with children, sleep too short. In addition, students who spend a lot of screen time for leisure go to bed later. In addition, one in four medical students sleeps too short and reports fairly poor to very poor sleep quality. This implies a need for education and intervention to raise awareness on screen use and sleep patterns and their association with performance and health among medical students.

These issues should be addressed during and within medical school and be used in interventions (behavioural but also situational preventive approaches). Raising awareness of the issues of screen use and sleep behaviour is important both for one's own health and performance in medical school—and later in the profession as an important contact person for patients.

Subsequent studies should investigate whether—and under what conditions—screen time is associated with poor sleep quality in medical students, and to what extent this influences performance, well-being and health.

### Supplementary Information

Supplementary Information.

## Data Availability

The datasets generated during the current study are available from the corresponding author on reasonable request.
