# Supplementary material for: Screen time and sleep among medical students in Germany
Source: Sci Rep. 2023 Sep 19;13:15462. doi: 10.1038/s41598-023-42039-8 (PMC10509232; doi:10.1038/s41598-023-42039-8)
Supplement: Supplementary file 1 — Supplementary Information. [file 41598_2023_42039_MOESM1_ESM.docx]

# Supplementary material

| **Supplementary material 1** Table cumulative bedtime | | | | |
| --- | --- | --- | --- | --- |
|  | Total | Gender | |  |
|  | n=415 | Male  n=114 | Female  n=292 |  |
| Bedtime, M (SD) | 23:11 (1.00) | 23:22 (1.00) | 23:07 (1.00) | p=0.025* |
| Bedtime until, n^a^ (%)^a^  22.00  23.00  0:00  1:00  2:00  3:00 | 63 (15.2)  247 (59.5)  364 (87.7)  398 (95.9)  412(99.3)  415 (100) | 11 (9.6)  57 (50)  94 (82.5)  110 (96.5)  112(99.1)  114 (100) | 50 (17.1)  184 (63.0)  262 (89.7)  279 (95.5)  290 (99.3)  292 (100) |  |
| M: average, SD: standard deviation  ^a^cumulated  *p<0.05 | | | |  |

| **Supplementary material 2** Table Sleep quality by gender |  |  | |
| --- | --- | --- | --- |
|  | Total | Gender | |
|  | n=414 | Male  n=114 | Female  n=291 |
| Very good, n (%) | 69 (16.7) | 20 (17.5) | 47 (16.2) |
| Fairly good, n (%) | 240 (58.0) | 67 (58.8) | 166 (57.0) |
| Fairly poor, n (%) | 95 (22.9) | 27 (23.7) | 68 (23.4) |
| Very poor, n (%) | 10 (2.4) | 0 (0) | 10 (3.4) |
|  | | | |
